# DNA damage induces a kinetochore-based ATM/ATR-independent SAC arrest unique to the first meiotic division in mouse oocytes

**DOI:** 10.1242/dev.153965

**Published:** 2017-10-01

**Authors:** Simon I. R. Lane, Stephanie L. Morgan, Tianyu Wu, Josie K. Collins, Julie A. Merriman, Elias ElInati, James M. Turner, Keith T. Jones

**Affiliations:** 1 Biological Sciences, Faculty of Natural and Environmental Sciences, University of Southampton, Southampton, SO17 1BJ, UK; 2 Sex Chromosome Biology Laboratory, The Francis Crick Institute, London, NW1 1AT, UK

**Keywords:** Chromosomes, DNA damage response, Etoposide, Oocyte, Meiosis, Spindle assembly checkpoint

## Abstract

Mouse oocytes carrying DNA damage arrest in meiosis I, thereby preventing creation of embryos with deleterious mutations. The arrest is dependent on activation of the spindle assembly checkpoint, which results in anaphase-promoting complex (APC) inhibition. However, little is understood about how this checkpoint is engaged following DNA damage. Here, we find that within minutes of DNA damage checkpoint proteins are assembled at the kinetochore, not at damage sites along chromosome arms, such that the APC is fully inhibited within 30 min. Despite this robust response, there is no measurable loss in k-fibres, or tension across the bivalent. Through pharmacological inhibition we observed that the response is dependent on Mps1 kinase, aurora kinase and Haspin. Using oocyte-specific knockouts we find the response does not require the DNA damage response kinases ATM or ATR. Furthermore, checkpoint activation does not occur in response to DNA damage in fully mature eggs during meiosis II, despite the divisions being separated by just a few hours. Therefore, mouse oocytes have a unique ability to sense DNA damage rapidly by activating the checkpoint at their kinetochores.

## Abstract (precis)

**Highlighted Article:** DNA damage in MI oocytes causes rapid recruitment and activation of spindle assembly checkpoint proteins at kinetochores leading to cell cycle arrest, thereby preventing the propagation of damaged chromatin.

## INTRODUCTION

The spindle assembly checkpoint (SAC) plays an essential role in reducing chromosome segregation errors by coupling anaphase-onset with biorientation, a state in which sister kinetochores are attached to microtubules emanating from opposite spindle poles (Foley and Kapoor, 2013; Lara-Gonzalez et al., 2012). Current models suggest that unattached kinetochores bind Mad1 protein (also known as Mad1l1), along with Mad2 (Mad2l1) to form a platform on which a conformational activation of a further recruited Mad2 can take place (Kulukian et al., 2009; Zhou et al., 2016). Activated Mad2 is then released into the cytoplasm to form part of a powerful inhibitor of the anaphase promoting complex (APC) known as the mitotic checkpoint complex (MCC) (De Antoni et al., 2005; Izawa and Pines, 2015; Kulukian et al., 2009). When chromosomes biorientate, Mad1 along with Mad2 is displaced from kinetochores. This leads to APC activation, through loss of the MCC, and so B-type cyclin and securin (Pttg1) degradation; these events are essential for mitotic exit (Foley and Kapoor, 2013; Lara-Gonzalez et al., 2012).

There is much interest in how the SAC is controlled during meiosis I (MI) in mammalian oocytes because of the high rates of mis-segregation of the paired homologous chromosomes (bivalents) during this division (Jones and Lane, 2013; Nagaoka et al., 2012; Touati and Wassmann, 2016). Such mis-segregation leads to early embryo loss, birth defects, and infertility. Despite these errors, the SAC is known to be present and active in mouse oocytes as loss or knockdown of its components increases rates of bivalent mis-segregation (Hached et al., 2011; Homer et al., 2005; Li et al., 2009; Niault et al., 2007; Touati et al., 2015; Zhang et al., 2005). However, although present, the SAC appears to be unable to respond to small numbers of non-biorientated bivalents (Gui and Homer, 2012; Hached et al., 2011; Jones and Lane, 2013; Kolano et al., 2012; Lane et al., 2012; Nagaoka et al., 2011). The reason why the meiotic SAC appears to be insensitive to a small number of errors is unclear, but might be related to the unique architecture of MI. It is a division of bivalents generating sister chromatid pairs with a single fused sister kinetochore achieving monopolar attachment.

The female meiotic SAC in oocytes is also activated by DNA damage associated with double strand breaks (DSBs) (Collins et al., 2015; Marangos et al., 2015). In these studies, both chemical (etoposide, bleomycin, phleomycin, doxorubicin) and physical (ionising radiation, UV-B) agents caused a metaphase arrest that was dependent on SAC activity. This contrasts with DNA damage induced during mitosis in somatic cells, which, although leading to segregation errors, is not associated with any capacity to activate the SAC and arrest cells (Bakhoum et al., 2014; Cesare, 2014; Giunta et al., 2010; Orthwein et al., 2014; Terasawa et al., 2014).

Here, we have studied the relationship between the canonical SAC, influenced by kinetochore microtubule attachment, and the DNA damage response (DDR)-induced SAC in mouse oocytes using 4D confocal laser scanning microscopy (4D-CLSM), pharmacological inhibitors and knockout mice. We find that the MI oocyte is uniquely sensitive to DNA damage, as arrest is not seen in eggs undergoing meiosis II (MII). This arrest does not require ATM or ATM kinases, and does not require a loss of tension or attachment between kinetochores and microtubules. Further, we demonstrate that the signal comes from kinetochores/centromeres and not chromosome arms.

## RESULTS

### Inhibition of APC activity associated with DNA damage in oocytes

DNA damage that occurs in fully grown germinal vesicle-stage oocytes immediately prior to nuclear envelope breakdown (NEB) causes an arrest several hours later (in MI) that is dependent on the SAC (Collins et al., 2015; Marangos et al., 2015). As such, the arrest is ameliorated by Mad2 knockdown, expression of a dominant-negative Bub1, or by inhibition of Mps1 kinase (also known as Ttk) activity. Here, we wanted to examine this association of DNA damage with SAC activation in more detail, and this was done by inducing DNA damage after NEB. This later addition of DNA-damaging agents allows the initiating event of DNA damage to be studied at the same time that the APC is active, and allows measurement of the extent to which the SAC is reactivated.

**Fig. 1.**
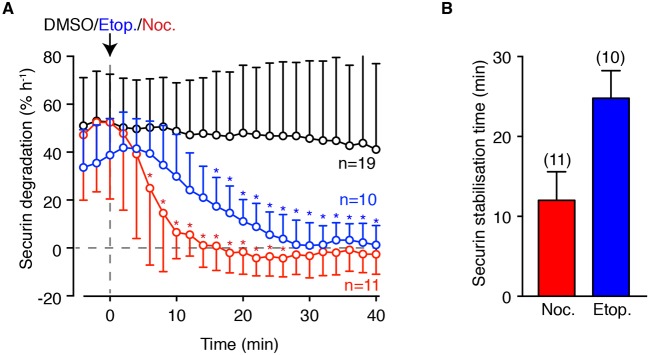
**DNA damage rapidly inactivates the APC during meiosis I in mouse oocytes.** (A) Degradation of securin-YFP fluorescence per unit time (% h^−1^) relative to the time of DMSO, etoposide or nocodazole. *P<0.05 (compared with time of drug addition; ANOVA with Dunnett's multiple comparison test). (B) Mean time from drug addition to stabilisation (i.e. net loss of fluorescence is zero) of securin-YFP, from data shown in A. Oocytes expressing securin-YFP were matured to 6 h after NEB. Error bars indicate s.d.

Initially, we wanted to determine how soon after DNA damage the SAC would be switched on sufficient to block APC activity. Oocytes expressing securin-YFP, as a real-time readout of APC activity, were imaged during addition of the canonical SAC activator nocodazole, or etoposide to induce DSBs. Oocytes were imaged between 6 and 7 h after NEB in order to record a measurable decline in securin levels, as a consequence of APC activity (Lane and Jones, 2014). As expected by its ability to depolymerise microtubules, nocodazole immediately diminished the rate of securin destruction and abolished it almost completely within a 10-15 min window (52.5±32.0% h^−1^ to 1.1±9.2% h^−1^, within 14 min; mean±s.d.; *n*=11; Fig. 1A,B). Similarly, etoposide also caused a substantial reduction in APC activity, although the rate of reduction was slower than with nocodazole (38.8±15.2% h^−1^ to 1.3±10.3% h^−1^, within 28 min; *n*=10; Fig. 1). Vehicle addition alone had no effect on securin degradation rate. Securin degradation first slowed significantly 6 min after nocodazole addition (*P*=0.0119, ANOVA), whereas following etoposide addition it took 14 min to achieve a significant reduction in the securin destruction rate (*P*=0.0085, ANOVA). This difference could reflect the indirect action of etoposide, which prevents repair of spontaneously generated DNA breaks. Most importantly, however, the timings illustrate the rapid response of the SAC to DNA damage in mouse oocytes, a process occurring over just a few minutes.

### Timing of DNA damage-induced recruitment of Mad1 to kinetochores

**Fig. 2.**
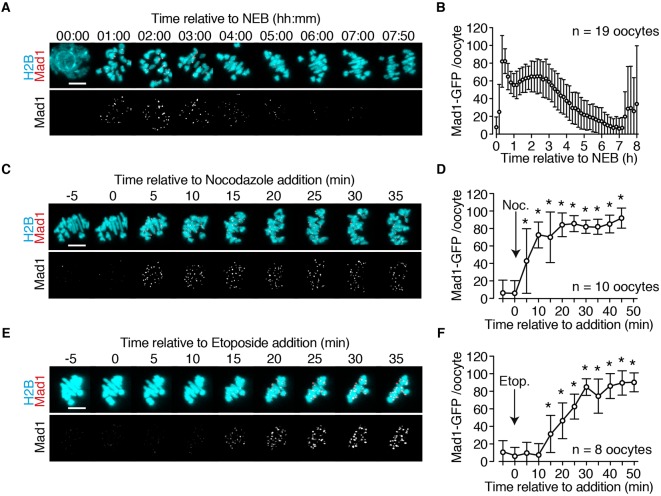
**Recruitment of Mad1 to bivalents following DNA damage.** (A) Representative images in oocytes expressing Mad1-2GFP and H2B-mCherry, tracked in MI by responsive time-lapse imaging. (B) Quantification of the bivalent-associated Mad1 signal shown in A. (C-F) Representative images (C,E) and quantification (D,F) of Mad1 signal on bivalents following addition of either nocodazole (C,D) or etoposide (E,F). Images captured at 5 min time points 6 h after NEB. (B,D,F) Data are normalised to the maximum signal. Scale bars: 10 µm. *P<0.0001 (compared with 0 min; ANOVA with Tukey's multiple comparisons test). Error bars indicate s.d.

The above findings suggest that DNA damage has a capacity to activate the SAC sufficiently such that APC activity is inhibited over the course of several minutes. Therefore, one would anticipate seeing activation of upstream components of the SAC within this time frame. Mad1-GFP was used as a dynamic probe of SAC activity during MI by microinjecting oocytes with its cRNA. In somatic cells, Mad1 loading onto, and then removal from, kinetochores is an essential step in switching on, and off, the SAC, respectively (Maldonado and Kapoor, 2011). Mad1-GFP has been used as a dynamic probe of SAC activity at kinetochores in a number of previous studies (Heinrich et al., 2014; Kruse et al., 2014; Matson and Stukenberg, 2014; Schweizer et al., 2013). In Mad1-expressing oocytes, an initial loading of this SAC component onto sister kinetochore pairs was observed shortly after NEB (Fig. 2A), and levels remained maximal for up to 3 h (Fig. 2B). Between 3 and 7 h after NEB there was a continual decline in kinetochore-bound Mad1 until the time of anaphase (Fig. 2A,B). As expected, this temporal profile of kinetochore-bound Mad1 correlates with APC activity, which begins to rise a few hours after NEB, and lasts for a period of ∼3-4 h (Lane and Jones, 2014; Lane et al., 2012).

The SAC, once satisfied, can still be reactivated both in mitosis (Clute and Pines, 1999; Waters et al., 1998) and meiosis (Lane et al., 2012). This SAC re-engagement could be observed as a significant rise in kinetochore-bound Mad1 following spindle disruption with nocodazole (Fig. 2C), but not using a vehicle control (Fig. S1A,B). This was achieved quickly, within 5 min following addition of this spindle poison (6.1±14.4 arbitrary units at 0 min versus 43.1±37.0 arbitrary units at 5 min, *P*<0.0001, ANOVA; Fig. 2D). The response to nocodazole was caused by activation of the SAC as kinetochore-bound Mad1 was quickly dissipated following addition of reversine, an inhibitor of the essential checkpoint component Mps1 kinase (Fig. S1C,D).

To determine whether DNA damage had the same temporal ability to recruit Mad1 onto kinetochores, the experiment was repeated with 40 µM etoposide, a dose effective at causing MI arrest (Collins et al., 2015). Similar to nocodazole, the checkpoint protein was observed to reload onto kinetochores (Fig. 2E) reaching significantly raised levels at 15 min following drug addition (6.1±10.0 arbitrary units at 0 min versus 31.4±21.1 arbitrary units at 15 min, *P*=0.0055, ANOVA; Fig. 2F). Mad1 levels continued to rise over the course of the next 15 min towards a steady-state high level.

### Aurora, Haspin and Mps1 kinase activity needed for DNA damage-induced arrest

**Fig. 3.**
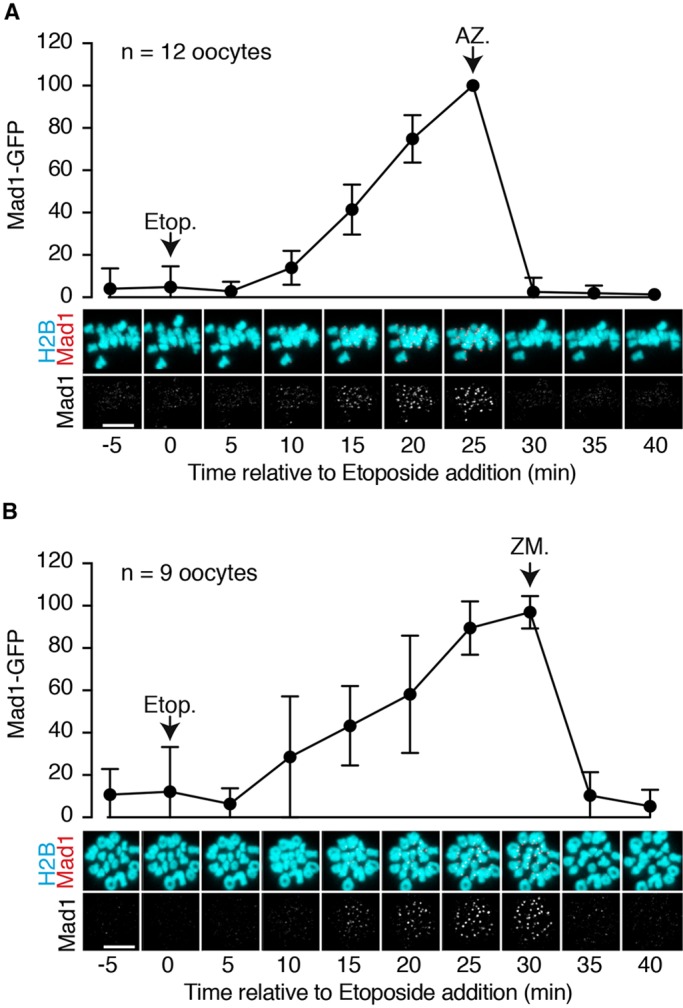
**Recruitment of Mad1 following DNA damage requires Mps1 and aurora kinase activity.** (A,B) Bivalents from oocytes expressing Mad1-GFP and H2B-mCherry were tracked in MI by responsive time-lapse imaging with images recorded every 5 min. Representative images showing Mad1 and H2B with etoposide addition, followed by either AZ3146 (A) or ZM447439 (B). The Mad1 signal is quantified above the corresponding images, normalised to the maximum signal. Scale bars: 10 µm. Error bars indicate s.d.

If DNA damage-induced SAC activation, acting at the kinetochore, were similar in pathway to that induced by spindle depolymerisation then it should be dependent on aurora and Mps1 kinases (Etemad and Kops, 2016; Musacchio, 2015). Activities of both kinases can be reduced pharmacologically, and have been used to ameliorate SAC activity (Ditchfield et al., 2003; Hewitt et al., 2010; Lane and Jones, 2014). We employed the aurora kinase inhibitor ZM447439 and the Mps1 inhibitor AZ3146, to block nocodazole-induced Mad1 recruitment to kinetochores in oocytes (not shown), and then examined their effects on DNA damage following etoposide. Both the Mps1 kinase inhibitor (Fig. 3A) and the aurora kinase inhibitor (Fig. 3B) were able to reverse the association of Mad1 with kinetochores, and this was rapid, being completed within 5 min of drug addition.

Haspin kinase (also known as germ cell-specific gene 2 protein; GSG2) recruits to centromeres, by phosphorylation of histone H3, the aurora kinase-containing chromosomal passenger complex (CPC) (Wang et al., 2010). Centromere-localised aurora kinase helps destabilise incorrect weak microtubule–kinetochore attachment, and is part of the SAC (Lara-Gonzalez et al., 2012; Musacchio, 2015). In mitosis, aurora B associates with the CPC but in oocytes such association is primarily observed with its meiotic homologue aurora C (Balboula and Schindler, 2014; Schindler et al., 2012). Interestingly, this meiotic aurora kinase is also found along chromosome arms and this localisation is blocked by 5-iodotubercidin (Balboula and Schindler, 2014; Nguyen et al., 2014; Quartuccio et al., 2017), a small-molecule inhibitor of Haspin kinase (De Antoni et al., 2012; Wang et al., 2012). Inhibition of Haspin kinase in oocytes is reported to reduce SAC activity (Quartuccio et al., 2017; Wang et al., 2016).

**Fig. 4.**
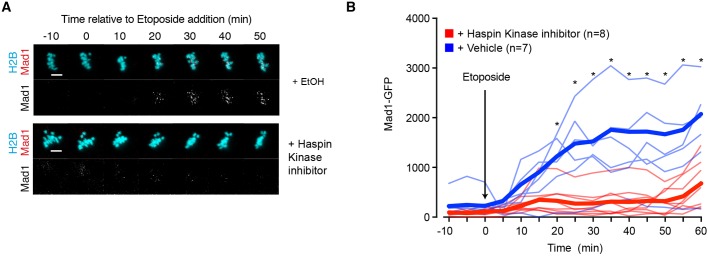
**Haspin kinase inhibition prevents Mad1 kinetochore recruitment following DNA damage.** (A) Representative images of oocytes 6 h after NEB expressing Mad1-GFP and H2B-mCherry. Addition of vehicle control (0.1% ethanol) or Haspin kinase inhibitor (0.1% 5-iodotubercidin) occurred prior to imaging. Both groups were also treated with 10 µM MG132 to prevent entry into anaphase. Etoposide was added at time 0. Scale bars: 10 µm. (B) Quantification of the Mad1 signal from the oocytes shown in A. Fine lines are individual traces and bold lines are mean data. *P<0.003 (Student's *t*-test).

We added the Haspin kinase inhibitor 5-iodotubercidin to oocytes before an etoposide challenge, and, as for ZM447439 and AZ3146, it also prevented Mad1 recruitment to kinetochores (Fig. 4). These data suggest that chromosome arm-localized aurora C/CPC senses the DNA damage and communicates this signal to trigger the SAC response.

### Mad1 and Cdc20 associate with kinetochores following DNA damage

**Fig. 5.**
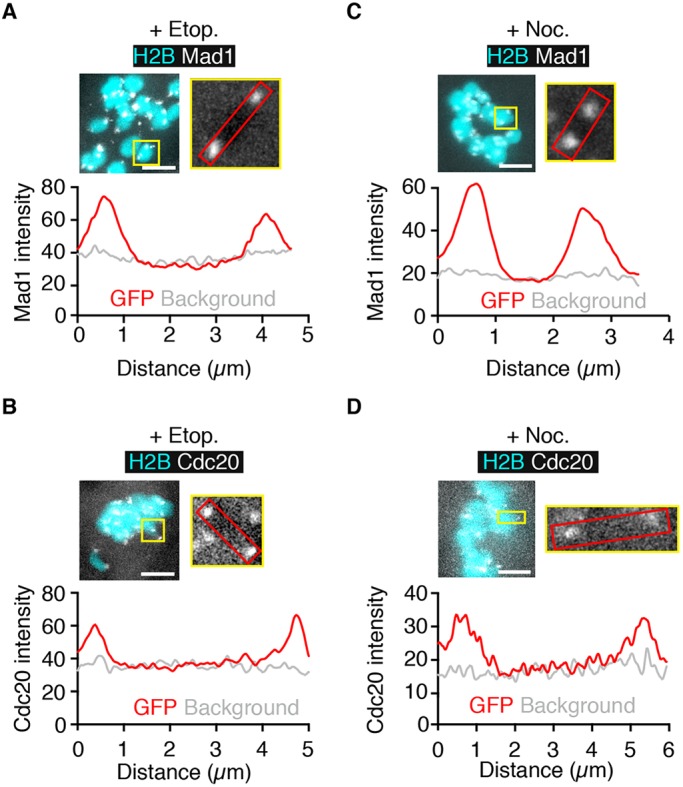
**SAC proteins form discrete foci at centromeres following DNA damage.** (A-D) Mad1-GFP (A,C) or Cdc20-GFP (B,D) fluorescence in oocytes co-expressing H2B-mCherry 1 h after addition of etoposide (A,B) or nocodazole (C,D). Images on the right show higher magnification of a representative bivalent (yellow box), for which Mad1 or Cdc20 intensity is plotted along the axial length of the bivalent in the graph below. Background readings were taken from a nearby area containing no chromosomes. For all plots Mad1 and Cdc20 fluorescence is only located in the centromeric region of the mouse telocentric bivalents. Scale bars: 5 µm.

Following etoposide-induced DNA damage, recruitment of Mad1 appeared to be restricted to kinetochores rather than the chromatin between the kinetochore pairs (Fig. 2E). Such a finding is consistent with our previous observation that Mad2 kinetochore levels are also raised following DNA damage (Collins et al., 2015), and that the Mad1-Mad2 complex is being recruited specifically to kinetochores. However, it has recently been observed in *Drosophila* dividing neuroblast cells that Cdc20/Fizzy, BubR1 and Bub3, but not Mad1 or Mad2, accumulate on chromosome arms following DNA damage (Derive et al., 2015). It may therefore be that some components of the SAC can be recruited to sites of DNA damage on chromosome arms whereas others are not. Hence, here we compared Cdc20 and Mad1 localisation to determine if any association with DNA could be visualised with either the canonical SAC activator nocodazole or with etoposide 60 min after treatment. Following nocodazole, as expected, recruitment of Mad1 (Fig. 5A) and Cdc20 (Fig. 5B) was confined to the two telocentric sister kinetochore pairs. Identical patterns of recruitment of Mad1 and Cdc20 were also observed following DNA damage (Fig. 5C,D). As a further precaution we exposed oocytes expressing Mad1-GFP to etoposide for 15 min, at a dose ten times higher than that used above. There was still no recruitment of GFP to the chromosome arms above background levels (Fig. S2). Therefore, no evidence was found for any Mad1 or Cdc20 localisation along the chromosome arms. If it does happen it is at a level not significantly above the background fluorescence, and is certainly far below the level of accumulation at kinetochores.

### DNA damage does not dissipate k-fibres or reduce bivalent stretch

**Fig. 6.**
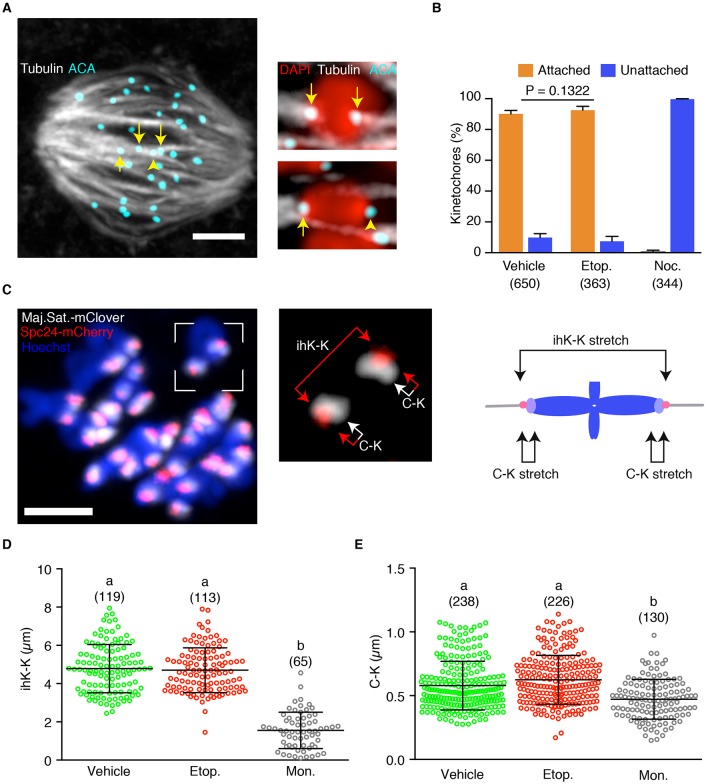
**DNA damage does not reduce kinetochore-microtubule attachment or tension.** (A) K-fibres immunostained for tubulin and anti-centromere antigen (ACA; counterstained with DAPI). Two bivalents are shown at higher magnification, showing examples of attached (arrows) and unattached (arrowhead) kinetochores. (B) Frequency of kinetochore attachment to k-fibres following addition of etoposide, nocodazole or vehicle (0.1% DMSO). Numbers of kinetochores assessed are shown in parentheses. Statistical test was Fisher's exact, error bars represent 95% confidence intervals. (C) Bivalents in an oocyte expressing Spc24-mCherry, a TALE protein against the major satellite repeat (Maj. Sat.-mClover), and counterstained with Hoechst; at 7 h after NEB. Right-hand image (detail of the boxed area on the left) and diagram show the measurements made on each bivalent: inter-homologue kinetochore stretch (ihK-K) and centromere-kinetochore stretch (C-K). (D,E) Measures of ihK-K (D) and C-K (E) following treatment with vehicle (DMSO, 0.1%), etoposide or monastrol (100 µM). Error bars indicate s.d.; number of measurements are in parentheses, different letters indicate statistically significant differences (*P*<0.05; ANOVA with Tukey's multiple comparison test). Error bars indicate s.d. Scale bars: 10 μm in A; 5 μm in C.

In the canonical SAC pathway the checkpoint responds to vacant kinetochores, using them as a template to generate the MCC (Foley and Kapoor, 2013; Kulukian et al., 2009; Lara-Gonzalez et al., 2012; Musacchio, 2015). Therefore, kinetochore attachment to microtubules was tested following DNA damage by measuring the percentage of end-on microtubule-attached kinetochores (k-fibres). They are associated with loss of SAC activity in mouse oocytes during MI (Lane et al., 2012; Rattani et al., 2013) and can be distinguished by their stability at cold temperatures (Amaro et al., 2010; Salmon and Segall, 1980; Toso et al., 2009). Therefore, following cold treatment and fixation, each kinetochore pair of a bivalent was assessed as being attached or unattached to k-fibres (Fig. 6A). In total, 44 oocytes at 7 h after NEB were imaged, with 1357/1760 (77.1%) kinetochores being successfully scored as attached or non-attached. In vehicle controls, the vast majority of kinetochores were associated with k-fibres (90.2%, *n*=650; Fig. 6B). Nocodazole, because it depolymerises tubulin, was very effective at severely reducing k-fibre number (0.3%, *n*=344; Fig. 6B), but, in contrast, etoposide had no effect (92.6%, *n*=363; Fig. 6B). These data suggest that the SAC is not being triggered by conspicuous k-fibre loss following DNA damage.

Tension generated across the kinetochore, in addition to k-fibre attachment, plays a role in switching off the SAC (Maresca and Salmon, 2009; Santaguida et al., 2011; Uchida et al., 2009). Tension development leading to stretch is propagated differently in MI, compared with MII and mitosis, because of the paired sister kinetochores and the structure of the bivalent (see Discussion). However, tension across the bivalent can be measured in two ways: (1) by the stretch across the length of the bivalent, or inter-homologue kinetochore stretch (ihK-K stretch), which would develop when amphitelic k-fibre attachment provides tension, and (2) centromeric-kinetochore stretch (C-K stretch), which would develop as the kinetochore-based k-fibre pulls on the centromere at one pole. To measure ihK-K and C-K stretch we overexpressed the outer-kinetochore protein Spc24 coupled to mCherry and a TALE protein that recognises the major satellite repeat (pericentromeric region) coupled to mClover (Fig. 6C). Spc24 is a kinetochore-based protein in mouse oocytes, as in all other cells, and is essential for correct bivalent segregation (Zhang et al., 2016), and the TALE construct has been used previously to label major satellite repeats in live mouse cells (Thanisch et al., 2014). Both measures of bivalent stretch were reduced when oocytes were treated with monastrol, showing biorientated bivalents decrease in length by ∼68% when k-fibre tension is lost (Fig. 6D), and that stretch between the centromere and the sister kinetochore pair is significantly reduced (Fig. 6E). However, treatment with etoposide had no effect on either of these measures of tension (Fig. 6D,E). Therefore, taken together in terms of the usual factors known to influence SAC activity and satisfaction, namely attachment and tension, no significant changes can be observed following DNA damage.

### ATM and ATR are not involved in DNA damage-induced arrest

The canonical sensing of DNA damage involves either ataxia telangiectasia mutated (ATM), or ATM and Rad3-related (ATR), which are phosphoinositide 3-kinase-related kinases (PIKKs) (Awasthi et al., 2015; Sirbu and Cortez, 2013). These two kinases are directly recruited to the site of DNA damage where they signal the process of repair (Caron et al., 2015; Falck et al., 2005; Nakada et al., 2003). Additionally, these PIKKs have also been implicated in the SAC, either interacting directly with specific SAC components or being involved in overall SAC efficacy (Dotiwala et al., 2010; Eliezer et al., 2014; Kim and Burke, 2008; Lawrence et al., 2015; Yang et al., 2014). However, a pharmacological ATM inhibitor failed to affect oocyte arrest when used previously on DNA damaged oocytes suggesting that instead ATR might be more important here (Marangos et al., 2015). Therefore, it was important to examine if ATM and ATR acting together were essential in transducing the DNA damage signal to the SAC.

**Fig. 7.**
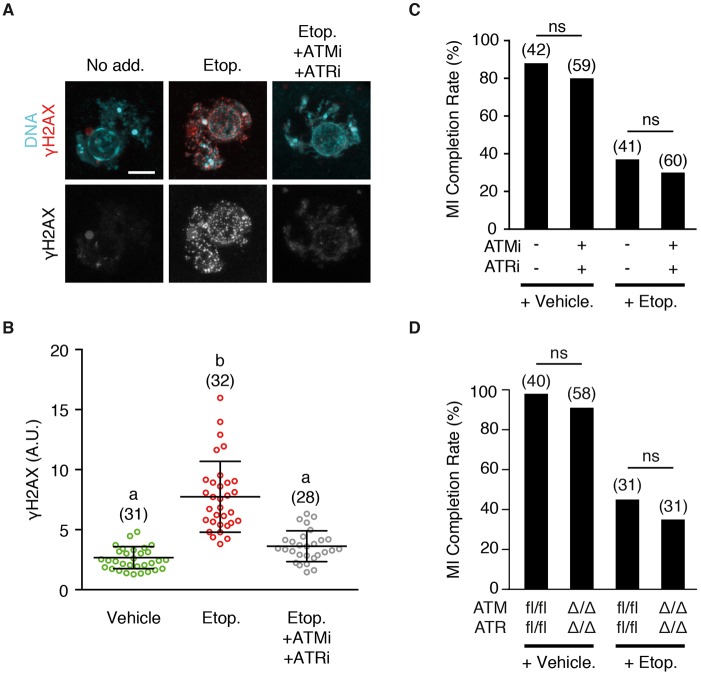
**Meiotic DNA damage-induced SAC activation is independent of ATM and ATR kinases.** (A) Representative γH2AX staining in the nuclei of oocytes before NEB, following addition of etoposide, or etoposide with ATMi (KU55933) and ATRi (ATR kinase inhibitor II). Scale bar: 5 µm. (B) Quantification of γH2AX levels as shown in A. Number of oocytes measured is shown in parentheses. Different letters indicate significant difference (*P*<0.0001; ANOVA with Dunn's multiple comparison test). (C) Percentage of oocytes completing MI following treatment with either etoposide or DMSO vehicle before NEB. Groups were matured in the presence or absence of ATMi and ATRi, and scored for polar body extrusion. (D) Oocytes from mice that were conditional double knockouts for ATR and ATM, or floxed littermate controls, were exposed to etoposide or a vehicle control, and assessed for completion of MI. (C,D) Number of oocytes used indicated in parentheses, statistical test used was Fisher's exact (ns, not significant). Error bars indicate s.d.

Inhibitors of ATM (KU55933) and ATR (ATR kinase inhibitor II) were used to inhibit γ-H2AX (histone 2AX phosphorylated on serine 139) staining, a marker of DNA double-strand breaks, following addition of etoposide (Fig. 7A,B, Fig. S3). Both PIKKs are involved in this process. In maturing oocytes, the presence of these PIKK inhibitors had no negative effect on the ability of the oocytes to complete MI. However, they failed to block the effects of etoposide on arrest (Fig. 7C). To confirm the lack of involvement of both ATM and ATR in the ability of DNA damage to cause SAC-mediated arrest, conditional double knockout mice were used. A conditional oocyte-specific knockout of these PIKKs was generated using mice with floxed genes for both *Atm* and *Atr*, mated to mice with *Cre* expression driven by the germ cell-specific promoter *Ddx4*. Oocytes of the double knockout, *Atm*^Δ/−^ *Atr*^Δ/−^, and of the control, *Atm*^fl*/−*^ *Atr*^fl/−^, were then challenged with etoposide before NEB, and allowed to mature *in vitro*. It was observed that the loss of both PIKKs had no impact on the ability of etoposide to cause an MI arrest (Fig. 7D), confirming the results from use of pharmacological inhibitors. It is concluded therefore that ATM and ATR are not essential for DNA damage induced by etoposide to cause metaphase I arrest.

### DNA damage to eggs does not block progression through MII

In oocytes during MI, DNA damage induced by etoposide appears to cause activation of the SAC at the kinetochores. This contrasts with somatic cells during mitosis, which demonstrate little ability to arrest their cell cycle in M phase following DNA damage. On completion of MI, oocytes have segregated their bivalents reductionally, and arrest spontaneously at metaphase II (metII). This second meiotic division (MII) involves equational division of pairs of sister chromatids, and thus resembles the division of chromosomes during mitosis (Clift and Schuh, 2013; Jones, 2011). We speculated whether these metII-arrested oocytes, in their sensitivity to DNA damage, resemble somatic cells during mitosis or oocytes during MI.

**Fig. 8.**
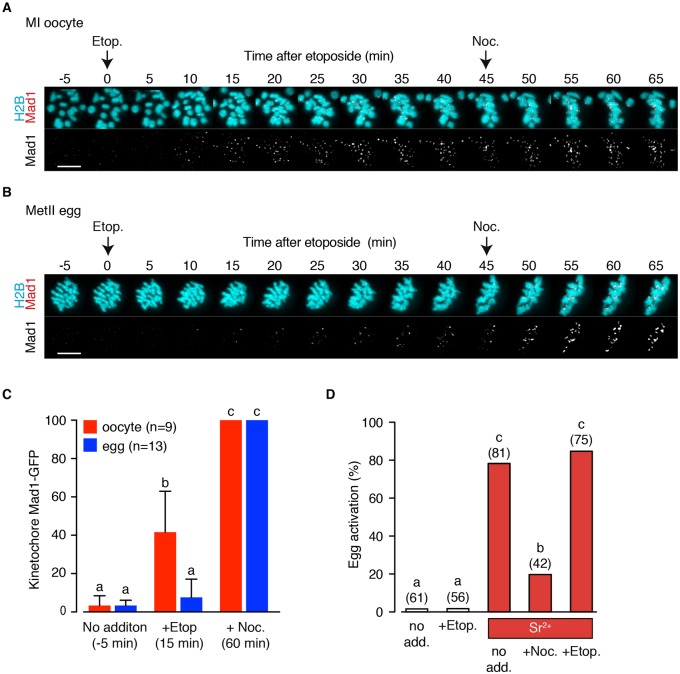
**DNA-damaged mature eggs can complete meiosis II.** (A,B) Representative time-lapse images of Mad1-GFP recruitment to the chromosomes of a meiosis I oocyte (A) and metII egg (B), following etoposide and nocodazole addition. Oocytes co-express H2B-mCherry. (C) Quantification of kinetochore-associated Mad1 after etoposide addition to oocytes or eggs. Fluorescence was normalised for each cell with respect to its maximal value following nocodazole addition. Error bars indicate s.d. Different letters indicate statistically different groups (*P*<0.0001, ANOVA with Tukey's multiple comparison test). (D) Parthenogenetic egg activation rates following either etoposide or nocodazole addition as indicated. Number of oocytes used indicated in parentheses; different letters indicate statistically different groups (*P*<0.005, Fisher's exact test with Bonferroni correction for multiple comparisons).

Mad1-GFP- and H2B-mCherry-expressing metII eggs were challenged sequentially with etoposide and then nocodazole in order to measure the increase in kinetochore-bound Mad1 following DNA damage relative to spindle depolymerisation. To make direct comparison with MI oocytes, while imaging on the stage of the confocal microscope we co-cultured MI oocytes and metII eggs, which could be exposed to drugs at the same time (Fig. 8A,B). Following etoposide addition we observed that compared with MI oocytes (Fig. 8A), metII eggs had a diminished response, attracting little Mad1 (Fig. 8B). After 45 min of exposure to etoposide, nocodazole was added to remove microtubules and reveal the maximal Mad1 signal in the respective groups. Because MI oocytes have twice as many kinetochores as MII eggs we did not compare the absolute values, but rather normalised each group against their own maxima (15 min after nocodazole addition). This revealed that in response to etoposide the metII eggs recruited a minimal amount of Mad1, whereas MI oocytes recruited a significant amount of Mad1 (Fig. 8C; *P*<0.0001, ANOVA with Tukey's multiple comparison test).

To determine whether this lack of response of metII eggs translated into an ability to be activated and so complete MII following DNA damage, etoposide-treated eggs were incubated with Sr^2+^-containing medium. MetII eggs cultured in this medium became activated, because of its ability to induce changes in intracellular calcium that mimic sperm (Bos-Mikich et al., 1997; Carvacho et al., 2013); and such activation could be blocked by nocodazole (Fig. 8D). This is predicted because physiological metII arrest is mediated by the APC inhibitor Emi2 (Fbxo43), but can also be induced by activation of the SAC (Madgwick et al., 2006; Tsurumi et al., 2004; Wu and Kornbluth, 2008).

DNA damage by itself did not cause any spontaneous egg activation, but, crucially, DNA-damaged eggs activated at a high rate in Sr^2+^-containing medium, at a level similar to eggs that were undamaged (Fig. 8D). To rule out the possibility that the differential effect of etoposide-induced DNA damage on meiotic arrest was due to the metII eggs having a lower permeability to etoposide, we used UV-B irradiation. UV-B (30 s exposure), like etoposide, is an effective activator of the SAC in meiosis I, causing MI arrest (Collins et al., 2015) (Fig. S4A). MetII eggs treated with the same dose of UV-B, activated at the same high rate as non-treated eggs (Fig. S4).

These findings clearly demonstrate that metII eggs behave the same as somatic cells in mitosis, in terms of their inability to arrest in M phase following DNA damage, and suggest that it is a unique feature of MI oocytes to activate the SAC and so arrest.

## DISCUSSION

We have shown here that DNA damage causes a rapid activation of the SAC during MI, allowing us to conduct an extensive examination of the mechanistic basis for this arrest. Previously, it had been observed that DNA damage induced before NEB would lead to a MI arrest (Collins et al., 2015; Marangos et al., 2015). The present findings extend this to show that damage does not have to be historically induced, but rather it has the capacity to halt ongoing oocyte maturation within minutes.

We found many spatiotemporal similarities to canonical SAC activation induced, for example, with spindle poisons. Firstly, it was the kinetochore, rather than sites of DSBs along the chromosome arms, that acted as the platform on which the SAC signal was generated. As such, both Mad1 and Cdc20 were observed to be quickly recruited to kinetochores following etoposide addition, without any noticeable change elsewhere on bivalents. This extends the previous observation that Mad2, Bub1 and BubR1 are also present on kinetochores following prophase I damage (Collins et al., 2015; Marangos et al., 2015). It suggests that the MI arresting mechanism following damage is the same regardless of when it is induced: before or after NEB. This conclusion is supported by the observation that both are dependent on Mps1 kinase activity (Collins et al., 2015; Marangos et al., 2015). These findings collectively point to a mechanism of SAC activation that is similar to the attachment/tension sensing mechanism in prometaphase of somatic cells (Etemad and Kops, 2016; Foley and Kapoor, 2013; Lara-Gonzalez et al., 2012; Musacchio, 2015). This is supported by the finding here that arrest appears to be sensitive to pharmacological aurora kinase inhibition. Furthermore, the higher-resolution imaging performed in the present study lends no evidence to the possibility that SAC components are localised along the chromosomes. Therefore, unlike in *Drosophila* dividing neuroblast cells, we cannot detect SAC proteins being recruited to the sites of DNA damage (Derive et al., 2015).

DNA-induced damage did not cause SAC activation during meiosis II, despite the fact that the two meiotic divisions are separated by only a few hours. However, eggs share the same property as somatic cells, which do not halt mitosis in response to damage, and instead respond in G1 by either repairing their DNA or undergoing apoptosis (Hustedt and Durocher, 2017). Therefore, on the basis of work presented here and what is known about the behaviour of somatic cells, it appears that DNA damage-induced SAC activation is only observed in MI.

**Fig. 9.**
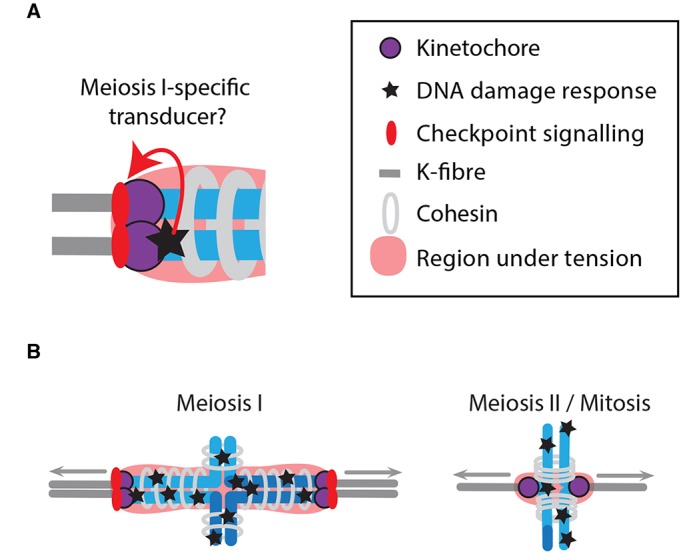
**Model to explain meiosis I-specific SAC arrest following DNA damage.** Possible mechanisms by which the SAC/DDR checkpoint functions specifically in meiosis I. (A) A meiosis I-specific protein could transduce a signal from sites of DNA damage in close proximity to the kinetochore to allow SAC signalling on the kinetochore; alternatively, this might happen due to the unique proximity of the two sister kinetochores in meiosis I. (B) The large volume of chromatin under tension during meiosis I might make the bivalent more sensitive to DNA damage compared with meiosis II or to mitosis.

Here, we give three possible explanations for the sensitivity of the MI oocyte to DNA damage. First, an MI-specific protein(s) might act as a transducer, propagating a DNA damage response in the vicinity of the kinetochore into a SAC signal (Fig. 9A). It may well prove that aurora kinase C, which can be found on chromosome arms in meiosis I due to Haspin kinase activity (Quartuccio et al., 2017), is involved in this process. Here, Haspin kinase inhibition did block DNA damage-induced Mad1 recruitment to kinetochores, and further work is needed to investigate this association. In support of its involvement is the loss of aurora C from chromosome arms in MII (Sharif et al., 2010; Shuda et al., 2009), a period here marked with loss of the ability of DNA damage to cause meiotic arrest. Additionally, kinetochore and pericentromeric chromatin appear to overlap in MI (see Fig. 6C), and other oocyte proteins do fulfil specific functions only in MI, such as Meikin and Emi1 (Kim et al., 2015; Marangos et al., 2007). Second, the unique chromatin architecture of the reductional division might endow the oocyte with sensitivity to DNA damage by virtue of the k-fibre pulling forces being spread across most of the length of the bivalent in MI (Fig. 9B) so accentuating the tension-sensing component of the SAC. However, this model is not preferred, as we did not detect any change in stretch across the bivalent or centromere following DNA damage. Therefore, if this mechanism is the case, the reduction in tension does not translate into changes we can measure here. Third, the sensitivity might be due to the unique structure of the co-segregating sister kinetochore pair in MI, which appears to allow both simultaneous k-fibre attachment and recruitment of SAC proteins (Brunet et al., 2003; Lane and Jones, 2014; Lane et al., 2012).

Historically, we and others have thought of the SAC in oocytes as being weak or ineffectual at detecting errors in bivalent biorientation (Gui and Homer, 2012; Jones and Lane, 2013; Kolano et al., 2012; Lane et al., 2012; Nagaoka et al., 2011; Sebestova et al., 2012; Touati and Wassmann, 2016). This is because several misaligned bivalents have no impact on the timing of progression through MI (Lane et al., 2012). This clearly, if extrapolated to humans, would help to explain the high rates of aneuploidy observed in human eggs and embryos. In contrast, no obvious morphometric changes are present in DNA-damaged oocytes that go on to show a robust meiosis I arrest. As such, it may be misplaced to describe the SAC as a weak or ineffectual checkpoint in oocytes. It provides an effective checkpoint with respect to DNA damage, and can respond to levels of DNA damage present in endometriosis (Hamdan et al., 2016). We propose that the SAC sensitivity to DNA damage comes as a result of the unique kinetochore or chromosome architecture of bivalents, likely involving meiosis-specific proteins. We present clear evidence here using knockout mice, that it does not involve the ATM and ATR DNA damage pathway. The existence of such a response in oocytes in MI provides a mechanism by which the formation of DNA-damaged embryos is prevented. It points to the SAC in meiosis being important for preventing not only whole chromosome aneuploidies but also the propagation of damaged chromatin.

## MATERIALS AND METHODS

### Reagents

All chemicals and reagents were from Sigma-Aldrich (UK) unless otherwise stated.

### Animals and oocyte culture

All mice were used in accordance with local and UK Home Office regulations on the use of animals in research. Three- to four-week-old C57Bl6 female mice (Charles River, UK) were used. Germinal vesicle-stage oocytes were released from the ovaries of hormonally primed females 44-52 h following intraperitoneal injection of 10 IU pregnant mares' serum gonadotrophin (Centaur Services, UK). M2 medium supplemented with milrinone (1 µM) was used for collection to maintain prophase arrest (Yun et al., 2014). Oocytes were mechanically stripped from surrounding cells. For maturation, oocytes were washed into fresh M2 media and cultured for ∼14-16 h.

For metII eggs, female mice were induced to superovulate by intraperitoneal injections of 10 IU pregnant mares' serum gonadotrophin, followed 48-52 h later by 10 IU human chorionic gonadotrophin (Centaur Services, UK). Cumulus oocyte complexes were collected 18 h later from the oviduct into M2 medium and briefly incubated in 300 IU ml^−1^ hyaluronidase (#H4272) to remove cumulus cells.

In some experiments, oocytes and eggs were exposed to 300 nm UV-B using a UV transilluminator (Hoefer Macrovue UV20; Fisher Scientific, UK) for a 30 s incubation time (Collins et al., 2015).

Double knockout mice for *Atr* and *Atm*, generated on a mixed genetic background of 129/Sv and MF1 strains, were produced by mating *Atr^+/−^ Atm^+/−^ Cre^tg/+^* males with *Atr*^flox/flox^ *Atm*^flox/flox^ females. *Atm*^flox/flox^ mice were obtained from the Jackson Laboratory, USA (strain 021444). The *Atm* flox allele contains two loxP sites flanking exons 57 and 58, which contain the core PIKK kinase domain of ATM. Cre-*lox*-mediated recombination of the *Atm* flox allele leads to the removal of these essential kinase domain-encoding exons (Callén et al., 2009). *Atr*^flox/flox^ mice were as described (Ruzankina et al., 2009).

### Inhibitors

Etoposide (40 µM) was added to oocytes for 15 min before NEB, or to maturing oocytes for the times as indicated. 5-Iodotubercidin (Cambridge Bioscience, CAY10010375) was dissolved in 100% ethanol and diluted in media at 1:1000 to give a working concentration of 0.5 µM. Other additions were nocodazole (400 nM); ZM447439 (10 µM; Bio-Techne, MN, USA), Mps1 inhibitor AZ3146 (2 µM; Bio-Techne), MG132 (10 µM), KU55933 (10 µM; Merck-Millipore, UK), bleomycin (1 µM; Abcam, UK) and ATR kinase inhibitor II (10 µM; Merck-Millipore, UK). All drugs were dissolved in DMSO and supplemented with the neutral detergent 200 µg ml^−1^ pluronic acid to aid dispersion, and used at dilutions of 0.1% or below.

### Parthenogenetic activation

Eggs were exposed to 0.1% DMSO, 40 µM etoposide or 400 nM nocodazole for 15 min. After washing, oocytes were either incubated in M2 for 6 h at 37°C or Ca^2+^-free M2 containing 10 mM SrCl_2_ (#439665) and 5 µg ml^−1^ cytochalasin B (#C6762) to make them diploid for 2.5 h followed by M2 for 3.5 h. Oocytes treated with nocodazole were exposed throughout the 6 h activation. Oocytes were fixed as described below, stained briefly in DAPI and examined for evidence of pronuclei.

### Kinetochore-microtubule attachment assay

Following 6 h maturation, oocytes were placed in ice-cold M2 medium for 4 min, fixed for 15 min in PBS containing 2% formaldehyde and 0.05% Triton X-100, and were then permeabilised for 15 min in PBS containing 0.05% Triton X-100 (Lane et al., 2012). Fixing and permeabilisation were performed at room temperature and oocytes were extensively washed with PBS between steps. Oocytes were incubated in a blocking buffer of 3% bovine serum albumin in PBS supplemented with Tween-20 and primary antibody for 1 h [anti-centromere antigen (ACA), Immunovision USA, #HCT-0100, 1:400] (Lane et al., 2012). Following several washes, oocytes were incubated with Alexa Fluor-555-conjugated secondary antibody (Life Technologies, UK, #A-21433, 1:500) and anti-α-tubulin-FITC (Sigma-Aldrich, UK, #F2168, 1:100) for 90 min. Antibody incubations were carried out at 37°C in blocking solution. Oocytes were briefly counterstained with DAPI (10 µg ml^−1^) to label chromatin before being mounted on glass slides with refractive index-matched Citifluor (#CFMAF1-10, Citifluor, UK).

### Homologue tension assay

Germinal vesicle-stage oocytes were microinjected with 500 and 600 ng µl^−1^ mRNA encoding Spc24-mCherry and TALE Major Satellite-mClover (Maj.Sat.-mClover; Addgene plasmid #47878, deposited by Maria-Elena Torres-Padilla; Miyanari et al., 2013; Thanisch et al., 2014), respectively. Spc24-mCherry was made by PCR from testis cDNA and restriction enzyme cloning into pRN3 derivative plasmid with C-terminal mCherry. Maj.Sat.-mClover binds directly to the major satellite repeat DNA sequence. Oocytes were matured in M2 media to metaphase, 7 h after NEB, and then counterstained with Hoechst (20 µg ml^−1^). Confocal image stacks were taken with a *z* separation of 300 nm and *x* and *y* pixel size of 0.036 µm. Images were processed in ImageJ (NIH, USA). A Gaussian blur (sigma=2) was applied and the centre of mass of each signal was determined using an in-house macro that utilised the Foci_Picker3D Plugin (Version 1.0, CAS, China) (Du et al., 2011). Data were exported to Excel (Microsoft, USA) and distances between foci were calculated using 3D Pythagoras.

### Immunofluorescence

Oocytes were fixed for 30 min in PHEM (PIPES, HEPES, EGTA, MgCl) buffer containing 2% formaldehyde and 0.05% Triton X-100, and were then permeabilised for 15 min in PBS containing 1% polyvinylpyrrolidone and 0.05% Triton X-100. Fixing and permeabilisation were performed at room temperature and oocytes were extensively washed with PBS buffer between solutions. Oocytes were incubated at 4°C overnight in a blocking buffer of 7% goat serum in PBS supplemented with Tween-20 before primary antibody incubation (rabbit anti-γH2AX, Abcam, #ab11174, 1:200; or rabbit anti-Mad2, 1:1000, a kind gift from Dr R. H. Chen, Taipei, Taiwan; and ACA, 1:400, #HCT-0100, Immunovision, USA). Following several washes, oocytes were incubated with goat anti-rabbit Alexa Fluor-633 and anti-human Alexa Fluor-555 secondary antibodies (Life Technologies, UK, #a-21070, 1:50). These incubations were carried out at 37°C in blocking solution. Oocytes were briefly counterstained with Hoechst (20 μg ml^−1^) to label chromatin before being mounted on glass slides with Citifluor (Citifluor, UK).

### cRNA manufacture

cRNA was transcribed *in vitro* from purified linear dsDNA templates. T7 or T3 mMessage RNA polymerase kits (Ambion, Life Technologies, UK) were used for *in vitro* transcription reaction (Lane et al., 2012). cRNA was suspended in nuclease-free water and the concentration of RNA products was determined by photospectroscopy.

### Microinjection

Microinjections into oocytes were performed on the stage of an inverted TE300 microscope (Nikon, Japan), using micromanipulators (Narishige, Japan) and a 37°C heated chamber (Intracel, UK) (Yun et al., 2014). A single injection with a 0.1-0.3% volume was achieved using timed injection on a Pneumatic Picopump (World Precision Instruments, UK) and pipette RNA concentrations of 100-1200 ng µl^−1^ (Lane et al., 2012; Yun et al., 2014).

### Immunofluorescence imaging

All images were acquired using a Leica SP8 fitted with hybrid detectors and 63× oil immersion lens. Fluorochromes were imaged sequentially. When quantifying levels of γH2AX, a *z* stack of the nuclear region was taken (∼30 µm) and acquisition settings were not altered throughout the experiment. γH2AX staining was calculated as total nuclear fluorescence, on an 8-bit scale, following background subtraction from a cytoplasmic region of equal area in the same oocyte. For the kinetochore-microtubule attachment assay, image stacks used a *z* resolution of 150 nm.

### Time-lapse imaging

Images were acquired at 2 or 5 min intervals using a Leica SP8 fitted with hybrid detectors, an environmental chamber set to 37°C, and a 63× oil immersion lens. Image stacks used a *z* resolution of 1.5 µm. In-lab software written in Python programming language was used to image multiple stage regions and to track up to 30 oocytes in experiments using H2B and Mad1-2GFP to ensure chromosomes stayed in the centre of a ∼26×26×24 µm imaging volume (Lane et al., 2017).

### Image processing

Time-lapse images from experiments with Mad1-2GFP were processed using ImageJ macros. The images were blurred (Gaussian blur, sigma=2) and background subtracted (subtraction of Gaussian blurred image, sigma=10). The histone channel was then used to make a binary image, which was dilated and used as a mask to filter out cytoplasmic Mad1 signals, leaving only Mad1 associated with the chromatin. A threshold was calculated such that two foci could be visualised on most bivalents at a suitable time point e.g. in prometaphase or after nocodazole addition. The 3D object counter plugin (Bolte and Cordelières, 2006) was then used to measure the volume of Mad1 associated with chromatin at each time point using the pre-determined threshold.

### Data analysis

All images were processed using ImageJ (NIH, USA) with extended functionality provided by in-house macros (Lane and Jones, 2014; Yun et al., 2014). For securin-YFP time-lapse experiments, fluorescence intensities (arbitrary units on an 8-bit scale) were recorded in ImageJ and subsequently analysed in Microsoft Excel.

### Statistical analysis

Dichotomous data were analysed by Fisher's exact test. Sample means were compared using either a Student's *t*-test or analysis of variance, with a post-hoc test as stated. GraphPad Prism software (GraphPad Software) was used for all tests.
